# Experimentation with psychoactive substances by public school students

**DOI:** 10.11606/S1518-8787.2017051006929

**Published:** 2017-08-25

**Authors:** Maria Eliane de Andrade, Igor Henrique Farias Santos, Antônio Araújo Menezes de Souza, Aliane Caroline Santos Silva, Tatiane dos Santos Leite, Cristiane Costa da Cunha Oliveira, Ricardo Luiz Cavalcanti de Albuquerque

**Affiliations:** I Programa de Pós-Graduação em Saúde e Ambiente. Universidade Tiradentes. Aracaju, SE, Brasil; II Departamento de Psicologia. Universidade Tiradentes. Aracaju, SE, Brasil; III Departamento de Enfermagem. Universidade Tiradentes. Aracaju, SE, Brasil

**Keywords:** Adolescent, Substance-Related Disorders, epidemiology, Risk Factors, Cross-Sectional Studies

## Abstract

**OBJECTIVE:**

To analyze the prevalence of exposure to psychoactive substances in public students of basic education and its association with sociodemographic characteristics.

**METHODS:**

This is a cross-sectional survey conducted from March to September 2015, involving 1,009 students of the basic and high school education in 20 public schools in the municipality of Aracaju, State of Sergipe, Brazil. The data have been compiled using questionnaires previously applied in national studies of the Brazilian Center for Psychotropic Drugs. The variables have been dichotomized for later logistic regression using the Chi-square test to analyze associations between experimentation with psychoactive substances and other sociodemographic variables; odds ratio and confidence intervals have also been calculated. The level of significance adopted was 5%.

**RESULTS:**

We have identified that 69.6% of the students have experimented alcohol and 12.4% cigarettes. Age (≥ 15 years) has shown a significant association with experimentation with alcohol (p < 0.001) and cigarettes (p = 0.02), acting as risk factor in both cases (OR = 2.34 and 1, 78, respectively), but it acted as a protective factor for the use of inhalants (p = 0.03 and OR = 0.58) and weight loss medication (p = 0.006 and OR = 0.44). Religious practice had a significant association with experimentation with alcohol (p = 0.01), functioning as a protective factor (OR = 0.56).

**CONCLUSIONS:**

We have concluded that the psychoactive substance most experienced by students was alcohol, followed by cigarettes, and chance for experimentation increases after the age of 15. Religious practice, in turn, acts as a protective factor for experimentation with alcohol.

## RESUMO

**OBJETIVO:**

Analisar a prevalência de exposição a substâncias psicoativas em estudantes do ensino básico de escolas públicas e sua associação com características sociodemográficas.

**MÉTODOS:**

Trata-se de um inquérito transversal realizado de março a setembro de 2015, envolvendo 1.009 alunos do ensino fundamental e médio em 20 escolas públicas de Aracaju, São Cristóvão e Nossa Senhora do Socorro. Os dados foram compilados por meio de questionários aplicados anteriormente em estudos nacionais do Centro Brasileiro de Drogas Psicotrópicas. As variáveis foram dicotomizadas para posterior regressão logística com aplicação do teste Qui-quadrado para analisar associações entre a experimentação de substâncias psicoativas e outras variáveis sociodemográficas, e calculada a razão de chances e seus intervalos de confiança. O nível de significância adotado foi de 5%.

**RESULTADOS:**

Identificamos que 69,6% dos estudantes têm experimentado álcool e 12,4% cigarro. A idade dos alunos (≥ 15 anos) mostrou associação significativa com a experimentação de álcool (p < 0,001) e cigarros (p = 0,02), atuando como fator de risco em ambos os casos (OR = 2,34 e 1,78, respectivamente), mas atuando como fator de proteção para o uso de inalantes (p = 0,03 e OR = 0,58) e remédios para emagrecer (p = 0,006 e OR = 0,44). A prática religiosa apresentou associação significativa com a experimentação de álcool (p = 0,01), funcionando como um fator de proteção (OR = 0,56).

**CONCLUSÕES:**

Conclui-se que a substância psicoativa mais experimentada pelos estudantes foi o álcool, seguida do cigarro, e que a chance de experimentação aumenta a partir dos 15 anos. A prática religiosa, por sua vez, atua como fator de proteção à experimentação do álcool.

## INTRODUCTION

The use of psychoactive substances by adolescents has been widely discussed in recent years as a public health issue, being this use mainly controlled by prevention measures^9^. The increase in the consumption of psychoactive substances among adolescents has caused concern in parents and educators, thus determining the search for new and efficient strategies of education, prevention, or combat of drug use^a^. Schools act as an ally in this process when this subject is related to the fundamental issues of the right to life and health^3^.

In view of the relevance of this topic, the World Health Organization, in partnership with the United Nations Children’s Fund, the United Nations Educational, Scientific, and Cultural Organization, and the Joint United Nations Program on HIV/AIDS with technical assistance from the Center for Disease Control and Prevention, has created an instrument to be applied in schools, mainly among students aged 13–17 years – the Global School-based Student Health Survey^b^. In this study, we have used this instrument to collect data, and we can compare our scientific findings with other national and international students regarding the prevalence of protective habits and factors related to the health of young persons in the school environment because the instrument is validated and standardized.

In Argentina, a survey with adolescents aged 13 to 15 years has indicated that alcohol use increased with age and most students had consumed alcohol in the past month. Those who consumed alcohol were more likely to have “poor mental health, use tobacco and drugs, have low involvement of parents in their lives, be dropouts, and suffer from bullying”^18^.

In Brazil, Carlini et al.^c^ have compared the last two national surveys (2004/2010) and found a decrease in the consumption of alcohol, inhalants, marijuana, anxiolytics, amphetamines, and crack cocaine, as well as an increase in cocaine use when asked about use at least once in the previous year. The most experimented substances were: mixture between alcohol and energetic drinks, anabolic steroids, ecstasy, and LSD.

In Sergipe, Brazil, a study conducted in Itabaiana, Aracaju, and Estância has shown that students use psychoactive substances, also within the school environment. The participants have affirmed the existence of the sale of illicit substances in the surroundings of educational institutions, making the adolescent vulnerable to situations also associated with violence^d^. Another study carried out in Aracaju has identified the use of licit substances by private school students in the urban area of the city, with a higher prevalence of alcohol consumption^6^.

Researchers have investigated possible influencing factors for the pattern of psychoactive substance use by adolescent students. They have identified that practicing a religion, having a good parental relationship^9^, receiving adequate information, living with parents, and having family habits (such as having at least one weekly meal with parents or guardians) are protective factors. As factors associated with substance use, we can mention the place where adolescents live^19^, lack of supervision by the guardians^20^, bad relationship with the parents and with oneself^9^, feeling loneliness, insomnia, and lack of friends^12^.

Rodrigues et al.^18^, after an intervention project with students, have identified that, despite the acceptance and learning acquired, there are other social factors that influence the consumption of substances and that are a burden for adolescents. Thus, they have concluded that, besides prevention practices in the school environment, this public needs a better quality of life, with access to quality health, courses, and sports and cultural activities, which would contribute to change the social reality of adolescents.

In this sense, a study was necessary to analyze experimentation with psychoactive substances among adolescent students. We believe that the results found can be compared with other scientific findings and, thus, public policies can be drawn up to improve the quality of life of adolescents. Therefore, we suggest the elaboration of strategies for health promotion and prevention, discussed and worked out by parents, educators, health professionals, and public security, in order to reduce the vulnerability of adolescents.

Given this scenario, the objective of this study has been to analyze the prevalence of exposure to psychoactive substances in public students of basic education and its association with some sociodemographic characteristics.

## METHODS

This is a cross-sectional survey conducted from March to September 2015, involving students aged from 10 to 24 years, from the state public basic and high school education in Great Aracaju, State of Sergipe, Brazil, in the municipalities of Aracaju, Nossa Senhora do Socorro, and São Cristóvão. In order to obtain the sample, we chose only the schools that had both basic education (eighth and ninth grade) and high school education (tenth to twelfth grade) in each selected municipality and that were recorded in the Education Portal of the State Secretariat of Education of Sergipe^e^. To define the size of the sample of students, we used the formula of Barbetta^2^, with distribution proportional to the number of students per school and grade. The total value of the minimum sample for basic education was 433, and for high school education, 427. We added 20% to the total sample to prevent impairment of the sample size after losses.

After approval of the project by the Ethics and Research Committee of the *Universidade Tiradentes* (Opinion 927.714), visits were made to the selected educational institutions to schedule and explain the study to the management of these institutions. Adolescents of both sexes who were present, agreed to participate, and returned the informed consent, signed in advance by their guardians when under 18 years of age, could participate in the study. We excluded adolescents whose enrollment records included some type of cognitive, emotional, auditory, or visual impairment, because of the possible need for an interpreter, which could compromise the confidentiality of the research.

For data collection, we applied the questionnaires used in Brazilian surveys conducted by the *Centro Brasileiro de Informações sobre Drogas Psicotrópicas* (Brazilian Center for Information on Psychotropic Drugs) adapted to Brazil by Carlini-Cotrin et al., Apud Carlini et al.^c^ The questionnaires contained closed and multiple-choice questions. Before giving the questionnaires, students were informed that filling was not mandatory, to allow them the freedom to return it blank or withdraw their consent at any time. The research team was trained regarding the time and form of application to ensure that everyone could make the collection homogeneously.

A researcher explained the objective of the study to the students in the classroom, without the presence of the teacher. The questionnaire was then distributed to them for self-completion. The adolescents were informed about the anonymity and secrecy of the information. The application took at most 30 minutes for basic education and one hour-lesson (50 minutes) for high school education. After answering, the questionnaire was returned to the researcher and placed inside an envelope, which was then properly sealed.

We removed from the sample the questionnaires that presented incoherence among answers or those in which the adolescents stated they had used a fictitious substance present in both instruments. The variables were labeled according to the questionnaire and subjected to descriptive analysis.

For the analysis of possible differences in the mean age of experimentation with psychoactive substances, the Kolmogorov-Smirnov test was used to assess normality and homoscedasticity. In the case of Gaussian distribution, Student’s t-test was used to compare the means of two groups, and ANOVA and Tukey’s test was used for comparison between three or more groups. When there was a non-Gaussian distribution, Kruskal-Wallis test and Dunn’s test were used to compare three or more groups of means, and the Mann-Whitney U test was used to compare two groups.

The frequency of experimentation with psychoactive substances was analyzed using the Chi-square test. To analyze associations between experimentation with these substances and the other sociodemographic variables, a dichotomization and logistic regression was performed using Chi-square test; in addition, odds ratio (OR) and their respective confidence intervals (CI) were calculated. For all the statistical tests described, we adopted a 95% confidence interval (95%CI) and a significance level of 5%. The analyses were done in the statistical program Statistical Package for the Social Sciences for Windows (SPSS) 16.0.

## RESULTS

This study was carried out in 20 public schools of Great Aracaju, located in the municipalities of Aracaju (80%), Nossa Senhora do Socorro (15%), and São Cristóvão (5%). A total of 1,009 adolescents participated, being them predominantly from high school and females. Mean age was 15.51 (SD = 1.58) years (Table 1).

**Table 1:** Sociodemographic characteristics of students of the public basic (8th and 9th grade) and high school education of Great Aracaju, State of Sergipe, Brazil, 2015.

Characteristic	n	%
Gender		
Male	419	41.5
Female	567	56.2
Not informed	23	2.3
Total	1,009	100
Age group (years)		
12–14	276	27.4
15–17	616	61.1
≥ 18	116	11.5
Not informed	1	0.1
Total	1,009	100
Education level		
Basic education	483	47.9
High school education	526	52.1
Total	1,009	100
Discrepancy		
Grade/age (years)		
None	447	44.3
1–2	456	45.2
≥ 3	105	10.4
Not informed	1	0.1
Total	1,009	100
Municipality		
Aracaju	627	62.1
Nossa Senhora do Socorro	270	26.8
São Cristóvão	112	11.1
Total	1,009	100

Regarding the means of initial age of experimentation for each substance, there was no significant difference for any substance analyzed in this study (p > 0.05). There was also no significant difference in relation to the sex of the participants and the initial age of experimentation.

Alcoholic beverages were the most experienced substance, followed by cigarettes and marijuana (Table 2).

**Table 2:** Consumption of substances by students of Great Aracaju, State of Sergipe, Brazil, 2015.

Substâncias psicoativas	Experimentation				

	Yes		No		p
	
	n	%	n	%	
Alcohol (n = 1,006)	702	69.8	304	30.2	< 0.001
Cigarette (n = 1,004)	125	12.5	879	87.5	< 0.001
Marijuana (n = 1,005)	85	8.5	920	91.5	< 0.001
Inhalants (n = 1,003)	81	8.0	922	92.0	< 0.001
Weight loss medication (n = 1,004)	54	5.4	950	94.6	< 0.001
Sedative (n = 1,000)	36	3.6	964	96.4	< 0.001
Cocaine (n = 1,002)	19	1.9	983	98.1	< 0.001

Among the variables studied (Table 3), age of the students (≥ 15 years) had a significant association with experimentation with alcohol (p < 0.001) and cigarettes (p = 0.02). There is a 2.34 times increase in the chance of this event happening for students aged 15 years and over. Regarding experimentation with cigarettes, the increase is 1.78. On the other hand, the entry in this same age group also showed a significant association with experimentation with inhalant substances and weight loss medication (p = 0.03 and p = 0.006, respectively). However, the increase in age groups acted as a protective factor for the use of these substances (odds ratio below 1.0). None of the other substances studied showed a significant association with the dichotomization of age group (p > 0.05).

**Table 3:** Logistic regression analysis on experimentation (use in life) with psychoactive substances and age group (dichotomized into <15 years and ≥ 15 years) of students of the public basic and high school education of Great Aracaju, State of Sergipe, Brazil, 2015.

Substance	< 15 years		≥ 15 years		Odds ratio	95%CI	p
	
	Yes	No	Yes	No			
Alcohol (n = 1,005)	154 (15.3%)	121 (12.0%)	547 (54.5%)	183 (18.2%)	2.34	1.75–3.14	< 0.001
Cigarette (n = 1,003)	23 (2.2%)	252 (25.1%)	102 (10.1%)	626 (61.8%)	1.78	1.11–2.87	0.02
Inhalants (n = 1,003)	31 (3.0%)	245 (24.4%)	50 (0.4%)	676 (67.3%)	0.58	0.36–0.93	0.03
Weight loss medication (n = 1,003)	24 (2.3%)	251 (25.0%)	30 (2.9%)	698 (69.5%)	0.44	0.25–0.78	0.006

We identified that the main psychoactive substances experimented by students were alcohol and cigarette, and the age group with the greatest chance of experimentation was the one of 15 years and over. As 15 years represents the initial age of entry into high school, this education level was used to analyze possible associations between other potential risk factors and the experimentation of said substances.

Regarding experimentation with alcohol, the variable of frequency of religious practices was significantly associated with experimentation with alcohol (p = 0.01) in the logistic regression analysis (Table 4). Thus, routine religious practices reduce the chance of experimentation with alcohol (OR = 0.56). The other variables studied in the logistic regression model were not significantly associated with the outcome studied.

**Table 4:** Logistic regression analysis between factors associated with experimentation with alcohol (use in life) among students of the public basic and high school education of Great Aracaju, State of Sergipe, Brazil, 2015.

Characteristic	Experimentation with alcohol (use in life)		Odds ratio	95%CI	p

	Yes	No			
	
	n (%)	n (%)			
Gender (n = 517)					
Female (ref.)	240 (46.4)	68 (13.2)	0.88	0.57–1.36	0.66
Male	167 (32.3)	42 (8.1)			
Parental relationship (n = 518)					
Live together (ref.)	191 (36.8)	60 (11.6)	1.05	0.69–1.61	0.88
Do not live together	217 (41.9)	50 (9.7)			
Physical activity (n = 521)					
Routine^a^ (ref.)	81 (15.5)	18 (3.5)	0.78	0.42–2.22	0.47
Sporadic or never	329 (63.1)	93 (17.9)			
Educational information (n = 522)					
Have received (ref.)	373 (71.5)	97 (18.5)	1.47	0.36–1.35	0.38
Have never received	38 (7.3)	14 (2.7)			
Religious practices (n = 520)					
Routine^b^ (ref.)	266 (51.1)	56 (10.7)	0.56	0.36–0.85	0.01
Sporadic or never	144 (27.7)	54 (10.4)			

ref.: variable of reference

^a^ At least 20 days/month.

^b^ Daily.

Regarding experimentation with cigarettes, we observed that parents living together is significantly associated with use in life, reducing the chance of experimentation for this substance (OR = 0.44), when compared to students whose parents do not live together (p < 0.001). The other variables analyzed in the logistic regression model had no significant association (p > 0.05) (Table 5).

**Table 5:** Logistic regression analysis between factors associated with experimentation with cigarettes (use in life) among students of the public basic and high school education of Great Aracaju, State of Sergipe, Brazil, 2015.

Characteristic	Experimentation with cigarettes (use in life)		Odds ratio	95%CI	p

	Yes	No			
	
	n (%)	n (%)			
Gender (n = 515)					
Female (ref.)	41 (7.9)	266 (51.6)	0.84	0.51–1.39	0.52
Male	32 (6.2)	176 (34.2)			
Parental relationship (n = 516)					
Live together (ref.)	22 (4.2)	229 (44.4)	0.44	0.27–0.71	< 0.001
Do not live together	52 (10.1)	213 (41.3)			
Physical activity (n = 519)					
Routine^a^ (ref.)	16 (3.1)	83 (16.0)	1.20	0.65–2.19	0.52
Sporadic or never	58 (11.2)	362 (69.7)			
Educational information (n = 520)					
Have received (ref.)	65 (12.6)	404 (77.4)	0.78	0.34–1.61	0.52
Have never received	9 (1.8)	42 (8.2)			
Religious practices (n = 518)					
Routine^b^ (ref.)	27 (5.2)	172 (33.2)	0.9	0.54–1.61	0.79
Sporadic or never	47 (9.1)	272 (52.5)			

ref.: variable of reference

^a^ At least 20 days/month.

^b^ Daily.

## DISCUSSION

In this study, most students under study were females, aged between 15 and 17 years, who attended high school. This sample characterization was similar to those reported in previous studies in Brazil^10^ ^,^ ^15^ and Spain^21^. In other studies^12^ ^,^ ^16^, the divergence in the profile of students may be associated with differences between the teaching types established in Brazil and in other parts of the world, which probably have different school curriculum.

The fact that most students had a percentage of discrepancy of one to two years in the school grade was also relevant. Few studies work on this data in their methodological approach, which makes it difficult to compare it with the literature and consequently give a more accurate interpretation. However, Galduróz et al.^9^, Oliveira et al.^15^, and Backes et al.^1^ have also identified a similar percentage of discrepancy in their respective studies. Therefore, these data suggest that this discrepancy seems to represent not only a characteristic of the sample used in this study, but a reality observed nationally. Studies carried by Ribeiro and Cacciamali^18^ have indicated that the problem of school discrepancy would be related to factors such as late school entry, school dropout, and grade repetition.

In this research, most students reported having experienced psychoactive substances at least once (“use in life”), and alcohol was the most mentioned, which corroborates previous work^5^ ^,^ ^7^ ^,^ ^13^. This result seems a worldwide reality, identified in Zambia and Uganda^20^, Portugal^14^, Spain^21^, Argentina^16^, and Mexico^22^. Thus, we can state that the consumption of alcoholic beverages by adolescents is a local, national, and international reality.

Another important data is the experimentation with alcohol by gender, which had no statistical significance. In the literature, national^4^ ^,^ ^10^ ^,^ ^14^ and international studies^16^, have also found similar results. Some studies have obtained different results as they have found significant difference in the use in life of alcohol by female respondents^12^ ^,^ ^15^.

In our study, the age of subjects (equal to or greater than 15 years) had a significant association with experimentation with alcohol, cigarettes, inhalants, and weight loss medication. However, the variable of age acted as a risk factor for alcohol and cigarette consumption, but surprisingly it acted as a protective factor for the use of inhalants and weight loss medication. Previous studies have stated that inhalants and weight loss medication do not appear as substances with significant results, which hinders the discussion of this result with other studies on the subject. Because they are licit substances, the use of alcohol and cigarettes may result from the easy access, lack of control in their sale to persons under 18 years of age, entry into the first job (adolescent trainee), and use by friends and family. On the other hand, the use of weight loss medication may be associated with the standard of beauty imposed by society, being reinforced by the need for self-affirmation in adolescence after the age of fifteen.

Among the substances mentioned, cigarette, marijuana, and weight loss medication are the most experienced ones, respectively. Villegas-Pantoja et al.^22^ have observed that, among psychoactive substances, the most consumed are alcohol and cigarettes. In addition, Brazilian studies have shown that the third most commonly used substance is marijuana^5^ ^,^ ^7^ ^,^ ^13^.

The aforementioned results may be associated with influencing factors, such as: sex, parental relationship, physical activity, educational information, and religious practices. In this study, the practice of sporadic or no religious activity was significantly associated with experimentation with alcohol. In a national study, Galduróz et al.^f^ has shown that adolescents who had religious practices used fewer psychoactive substances in the Brazilian Northeast region. This may be due to the fact that this region traditionally maintains religious habits, which over time have become social and cultural habits. On the other hand, festivals considered as profane attract young persons who have less identification with any religion, being a place conducive to the consumption of psychoactive substances^3^.

Among the factors associated with the use of psychoactive substances, parents who do not live together was associated with experimentation of cigarettes. Other research studies have also presented parental relationship as an influencing factor in the consumption of this and other psychoactive substances mentioned in this study. We can mention as protective factors: having a good relationship with parents and oneself, living with parents, having simple family habits, and parents’ knowledge of what adolescents do with their free time^4^ ^,^ ^9^ ^,^ ^12^. Parents need to work, and because they do not have anyone to leave their children with, young individuals are left alone and thus spend most of their free time without supervision.

In this study, most students reported receiving educational information on the use of psychoactive substances, but there was no significant association between these variables. This result differs from those found in the literature, in which authors have stated that no educational information about the use of psychoactive substances would be an important influencing agent for their consumption^19^ ^,^ ^22^. In Brazil, the media presents the use of alcohol in attractive advertisements, leaving adolescents vulnerable to consumption, although alcohol can be only sold to those over 18 years of age^8^. In addition, access to these substances is facilitated as they are offered by family and friends^5^ ^,^ ^11^ ^,^ ^14^. The information offered to the students does not seem to have been enough to stimulate new behaviors.

On the other hand, we need to consider that several variables analyzed in this study had no significant associations with experimentation with psychoactive substances. Such a result, however, may be a reflection of the small sample size, as suggested by the very broad confidence intervals, which seems to entail the need for further studies with larger samples.

As they are present everywhere, including the surroundings of schools, psychoactive substances can interfere with the daily life and social relations of adolescents. The school environment is a space conducive to the socialization of individuals. Institutions should be prepared to handle situations also associated with the use of these substances, as they may interfere in the teaching-learning process. In this context, we suggest that intervention studies should be carried out in the school environment, since it is crucial to draw up health education strategies aimed at guiding the school community about the conceptions of the use of psychoactive substances and the consequences arising from this use.

## Footnotes

a Ribeiro WA. Abordagens pedagógicas de prevenção do uso indevido de drogas por adolescentes: da prática da opressão à prática da liberdade [dissertation]. Belo Horizonte: Pontifícia Universidade Católica de Minas Gerais; 2001 [cited 2015 Oct 10]. Available from: http://www.bdae.org.br/dspace/bitstream/123456789/1872/1/tese.pdf

b Centers for Disease Control and Prevention. Epi Info. Atlanta: CDC; 2002 [cited 2015 Oct 10]. Available from: https://www.cdc.gov/epiinfo/index.html

c Carlini ELA, Noto AR, Sanchez ZM, Carlini CMA, Locatelli DP, Abeid LR, et al. VI Levantamento nacional sobre o consumo de drogas entre estudantes do ensino fundamental e médio da rede pública e privada de ensino nas 27 capitais brasileiras. São Paulo: CEBRID-Centro Brasileiro de Informações dobre Drogas Psicotrópicas da UNIFESP; Brasília (DF): SENAD-Secretaria Nacional de Políticas sobre Drogas; 2010 [cited 2015 Oct 10]. Available from: http://www.antidrogas.com.br/downloads/vi_levantamento.pdf

d Sergipe, Secretaria do Estado da Educação. Diagnóstico referente ao uso de drogas nas escolas públicas estaduais de Sergipe: situação preliminar. Aracaju: SEED-SE; 2010.

e Sergipe, Secretaria do Estado da Educação de Sergipe. Portal da Educação. Aracaju; s.d. [cited 2014 Jun 9].

f Galduróz JCF, Noto AR, Fonseca AM, Carlini EA. V levantamento nacional sobre o consumo de drogas psicotrópicas entre estudantes do ensino fundamental e médio da rede pública de ensino nas 27 capitais brasileiras 2004. São Paulo: Universidade Federal de São Paulo, CEBRID; 2004 [cited 2017 Jun 20]. Available from: http://www2.unifesp.br/dpsicobio/cebrid/levantamento_brasil2/
